# Supplementary material for: Novel Pathogenic Mutations Identified from Whole-Genome Sequencing in Unsolved Cases of Patients Affected with Inherited Retinal Diseases
Source: Genes (Basel). 2023 Feb 9;14(2):447. doi: 10.3390/genes14020447 (PMC9956865; doi:10.3390/genes14020447)
Supplement: Supplementary file 1 [file genes-14-00447-s001.zip › genes-2193605-supplementary.pdf]

Supplementary Table S1: SpliceAI prediction scores of novel splicing variants.

chromosomal position	DS_AG	DS_AL	DS_DG	DS_DL	DP_AG	DP_AL	DP_DG	DP_DL
chr6:65704184:C>A	0.65	0.02	0.95	0.02	60	214	2	125
chrX:85237775:C>G	0.68	0.04	0.58	0	-3	-1	-117	435
DS_AG, delta score of acceptor gain; DS_AL, delta score of acceptor loss; DS_DG, delta score of donor gain; DS_DL, delta score of donor loss; DP_AG, delta position of acceptor gain site relative to the mutation site; DP_AL, delta position of acceptor loss site relative to the mutation site; DP_DG, delta position of donor gain site relative to the mutation site; DP_DG, delta position of donor loss site relative to the mutation site.								

Supplementary Table S2: Primer sequences for RT-PCR.

MEP-123-F	tgggaagcgaaagggtcgacACATTTTTATATTTACAGCA
MEP-123-R	ggcagctcgactagtctagaAAGGAAAAGAAGAAAACTGG
MEP-395-F	tgggaagcgaaagggtcgacTAATTGTTTAAAAACCTTTC
MEP-395-R	ggcagctcgactagtctagaCATATTGGGGCTCCGGATCC

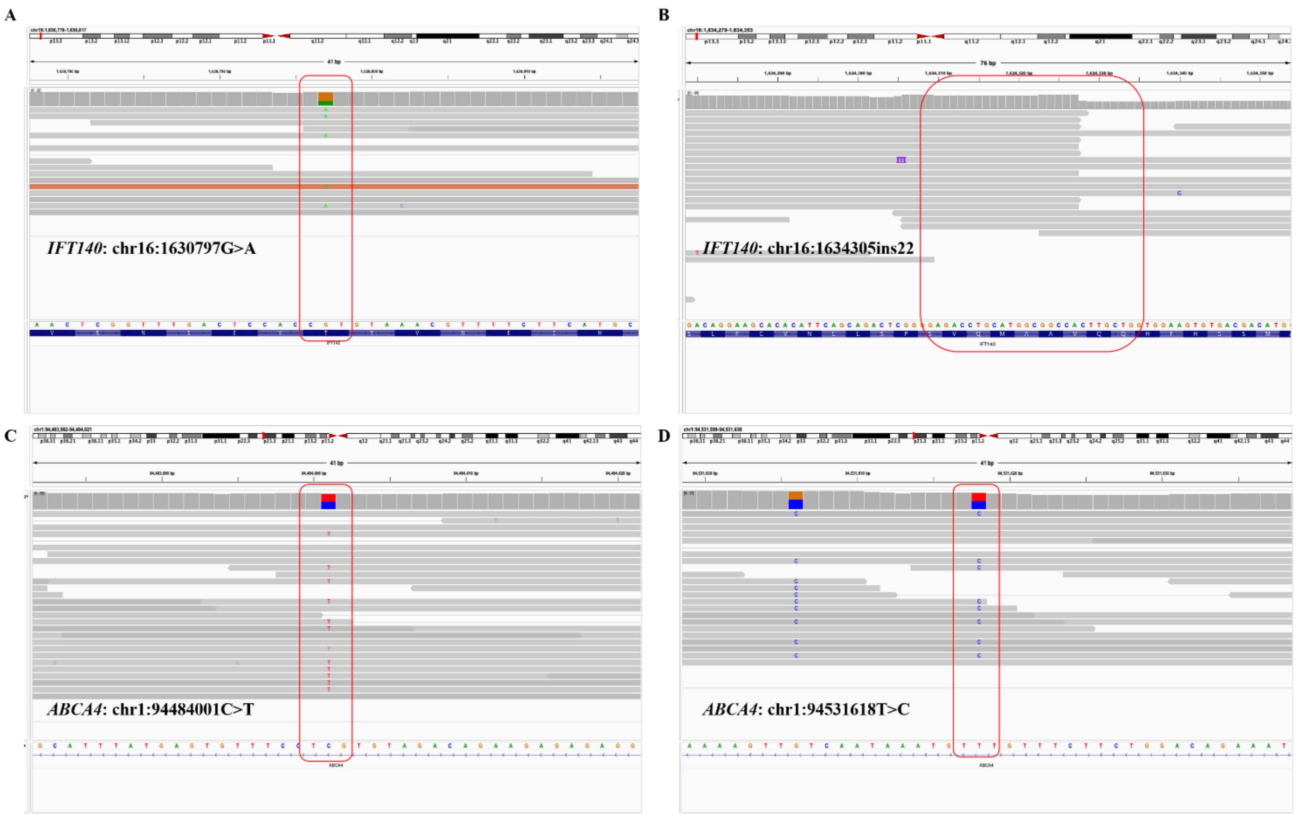

Figure S1: IGV plots of probands affected with IRD: (A,B) IGV plots of MEP-398 in *IFT140* (chr16:1630797G>A & chr16:1634305ins22) and (C-D) IGV plots of MEP-082 in gene *ABCA4*: (chr1:94484001C>T and chr1:94531618T>C);

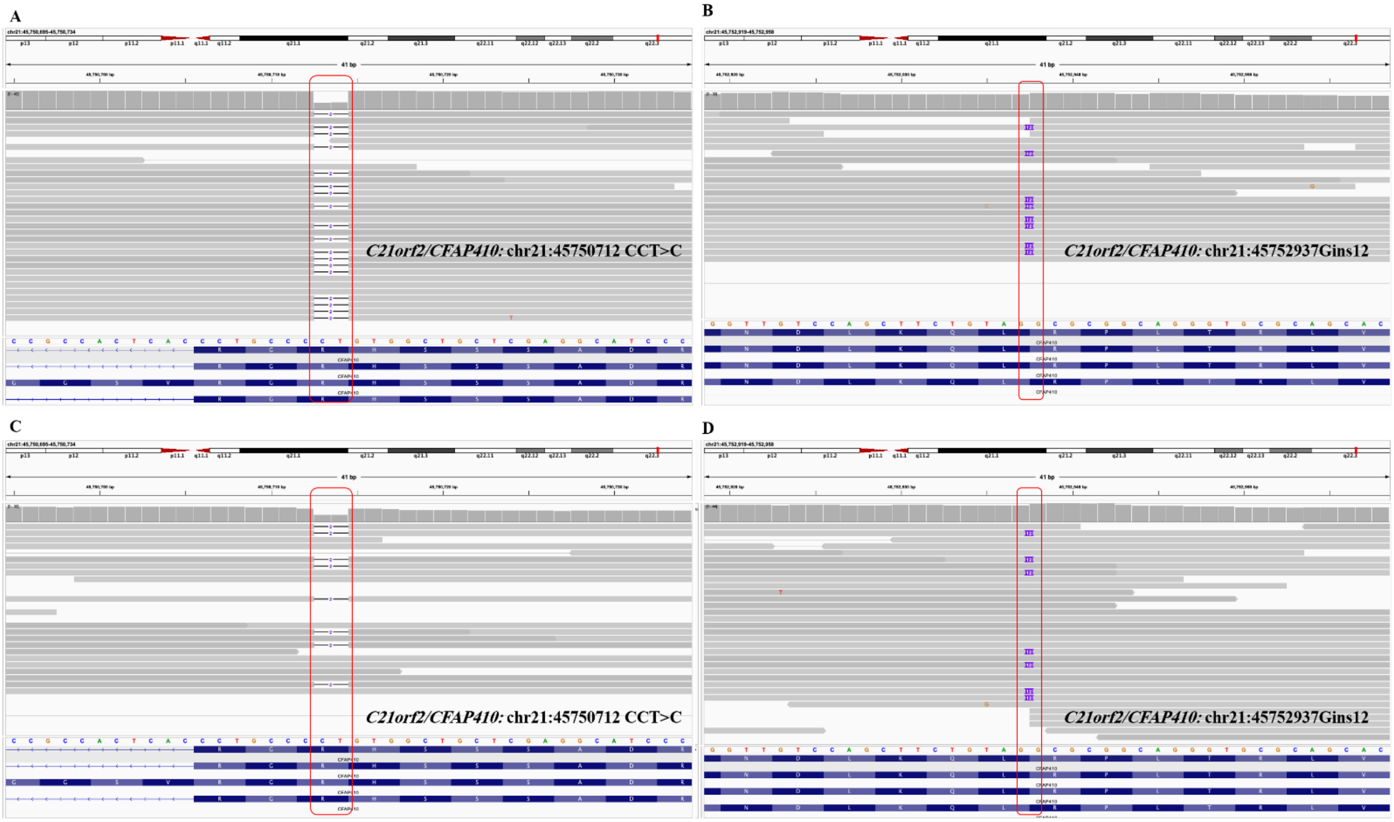

Figure S2: . IGV plots of siblings affected with IRD: (A-B) IGV plots of MEP-662 in gene *C21orf2/CFAP410* (chr21:45750712 CCT>C and chr21:45752937Gins12) and (C-D) IGV plots of MEP-663 in gene *C21orf2/CFAP410* (chr21:45750712 CCT>C and chr21:45752937Gins12)
